# Analysis of *Streptococcus pneumoniae* using Fourier-transformed infrared spectroscopy allows prediction of capsular serotype

**DOI:** 10.1007/s10096-019-03622-y

**Published:** 2019-07-09

**Authors:** Irene Burckhardt, Kerstin Sebastian, Norman Mauder, Markus Kostrzewa, Florian Burckhardt, Stefan Zimmermann

**Affiliations:** 1 Department for Infectious Diseases, Microbiology and Hygiene, University Hospital of Heidelberg, Im Neuenheimer Feld 324, 69120 Heidelberg, Germany; 2 Bruker Daltonics, Bremen, Germany; 3 Epiet Alumni Network, Heidelberg, Germany

**Keywords:** FT-IRS, Pneumococcus, Capsular typing, Vaccine serotypes, PCV13, PSV23

## Abstract

Determination of the capsule type of clinical isolates of *Streptococcus pneumoniae* is a prerequisite for epidemiological studies and further vaccine development. The Quellung reaction for serotyping is expensive and mostly done in reference centres. We wanted to evaluate whether Fourier-transformed infrared (FT-IR) spectroscopy is suitable for capsular type analysis and prediction of pneumococcal serotypes. We used the IR-Biotyper™ (Bruker) to create a database containing the spectra of 120 strains from invasive disease. The strains covered the 24 vaccine serotypes contained in the 13-valent conjugate vaccine (PCV13) and the 23-valent polysaccharide vaccine (PSV23). Hierarchical clustering analysis was performed. Finally, two different classification sets were created (PCV13 and PSV23). They were used to predict the serotype of 168 different challenge strains (invasive and non-invasive disease) covering 48 different serotypes (vaccine and non-vaccine types). FT-IR spectra from pneumococci (1300–800 cm^−1^) clustered along their serotype as determined by the Quellung reaction (120 strains, 24 different serotypes). Strains with unknown serotype fell within the cluster of the correct serotype, as long as the latter was represented in the database (168 strains, 48 different serotypes). Concordance between the Quellung reaction and FT-IR spectroscopy was excellent (kappa ≥ 0.75). FT-IR spectroscopy is a fast and cost-effective method to predict the capsular serotype of pneumococci.

**Electronic supplementary material:**

The online version of this article (10.1007/s10096-019-03622-y) contains supplementary material, which is available to authorized users.

## Introduction

The capsule of *Streptococcus pneumoniae* is a significant pathogenicity factor, which protects pneumococci from phagocytosis and has an important role during colonization and invasive disease [1–3]. The propensity for invasion of pneumococci correlates with the capsular serotype [4, 5] and host defence against pneumococci is based on serotype-specific opsonophagocytosis. Additionally, there are reports on the influence of serotype on disease development and outcome [6–9]. More than 90 different serotypes are currently described [10] and the commercially available pneumococcal vaccines contain up to 23 different capsule polysaccharides. The reference method for serotyping is the Quellung reaction. SSI Diagnostica (formerly part of the Statens Serum Institute, Denmark) provides the necessary pool, type, group and factor sera according to the Danish system of pneumococcal serotyping [11]. However, this technique is expensive and time consuming. Therefore, it is only available at specialized reference centres.

Fourier-transformed infrared spectroscopy (FT-IRS) uses infrared light to create spectra from whole bacterial cells. The IR-spectrum is dependent on the chemical bonds within the bacterium [12]. The section between 1200 and 900 cm^−1^ was initially called the “polysaccharide region” [13]. FT-IRS was used for streptococcal species identification (1300–900 cm^−1^) [14] and for discriminating pneumococcal serotypes (1200–900 cm^−1^) [15, 16]. Vaz and co-workers were able to correctly discriminate 4 serogroups (serogroup 9, 14, 19 and 23). Serotype discrimination was possible within serogroup 9 (9N and 9V) and 19 (19A and 19F) but difficult within serogroup 23 (23A, 23B, 23F). We wanted to investigate whether it is possible to discriminate between all serotypes of the PCV13 and PSV23 vaccines with FT-IRS and to predict the correct serotypes of strains with unknown serotype.

## Methods

### Hardware

For all experiments, an IR-Biotyper™ (Bruker Daltonics, Bremen, Germany) was used with a 96-spot silicon microtiter plate. The following controls were included in each run: (a) Bruker Infrared Test Standards (IRTS), each of the two standards was applied twice per target and run (hardware control), and (b) *S. pneumoniae* ATCC 49619, fresh 24-h culture on Columbia 5% sheep blood agar (BD, Heidelberg Germany), grown at 36 °C in 5%CO_2_ for 24 h and applied in triplicate (in process control).

### Bacteria

Throughout the study, only *S. pneumoniae* strains were used as confirmed by optochin susceptibility (BD BBL Taxo P Discs) on MHF-agar (Mueller Hinton agar + 5% horse blood + 20 mg/l β-NAD, BioMérieux, Nürtingen, Germany), incubated at 36 °C in 5% CO_2_ [17].

All strains for spectra generation were grown on Columbia Agar 5% sheep blood (Becton Dickinson), for 24 h at 36 °C and 5% CO_2_. Bacteria were directly applied to the target with a 1-μl plastic loop. The target was left to dry on a 40 °C heating block for 10 min before spectra acquisition. Each spot was measured once.

For database generation, each strain was applied onto 5 different spots (technical replicates) on three following days (biological replicates) with new 24-h subcultures to account for technical variability. Only spectra with valid target controls were used for database generation. The aim was to include at least 12 of 15 different spectra of each strain.

Challenge strains were applied onto 3 different spots on a single day.

All pneumococcal strains used for database creation and classification dataset creation had been previously serotyped (Quellung reaction). After thawing of each strain, its serotype was confirmed by a serotype-specific PCR before the isolate was used for database generation [18, 19].

The serotypes of all strains used for the database challenge/prediction of serotype were determined using the Quellung reaction. In case of no match or mismatch of the FT-IR spectrum with the Quellung result, the serotype was confirmed by PCR.

### Database generation and classification set definition

The 24 serotypes used for database generation were chosen according to the serotypes included in PCV13 (Pfizer) and PSV23 (Sanofi Pasteur MSD): the PCV13 classification set included the following serotypes: 1, 3, 4, 5, 6A, 6B, 7F, 9V, 14, 18C, 19A, 19F, 23F; the PSV23 classification set additionally included the following serotypes: 2, 8, 9N, 10A, 11A, 12F, 15B, 17F, 20, 22F, 33F. (remark: the database PSV23 contained 24 serotypes because both serotypes from serogroup 6 (6A and B) were included; the PSV23 vaccine contains only serotype 6B).

For each serotype, 5 strains were randomly chosen from the strain collection of the department or provided by the German National Reference Centre for Streptococci, Aachen, Germany (GNRCS, Dr. M. v.d. Linden). All strains originated from bacteraemia, collected and serotyped (2006–2017) within the scope of the nationwide non-mandatory surveillance of invasive pneumococcal disease in Germany (“Pneumoweb”) [20].

### Challenge of database

The strains used for challenging the database were part of the strain collection of the Department for Infectious Diseases, Microbiology and Hygiene, University of Heidelberg, Germany. Originally, they had been isolated from patient material and identified to species level and serotyped at the GNRCS (2006–2018). For a detailed list of strains, see Table 2 and supplementary Table 1. Invasive as well as non-invasive isolates were used (see Table 1).

**Table 1:** Specimen types of challenge strains

Specimen type	*n*
Blood culture	108
Respiratory tract (sputum, bronchial secretion, lavage)	40
Cerebrospinal fluid	15
Middle ear fluid	3
Abdominal swab (not further specified)	2
Total	168

### Clustering

Hierarchical cluster analysis was done using the Euclidean—average—mean spectra algorithm, which is included in the software of the IR-Biotyper (version 1.5). Dendrogrammes of the PCV13 and PSV23 classification set were generated.

**Fig. 1:**
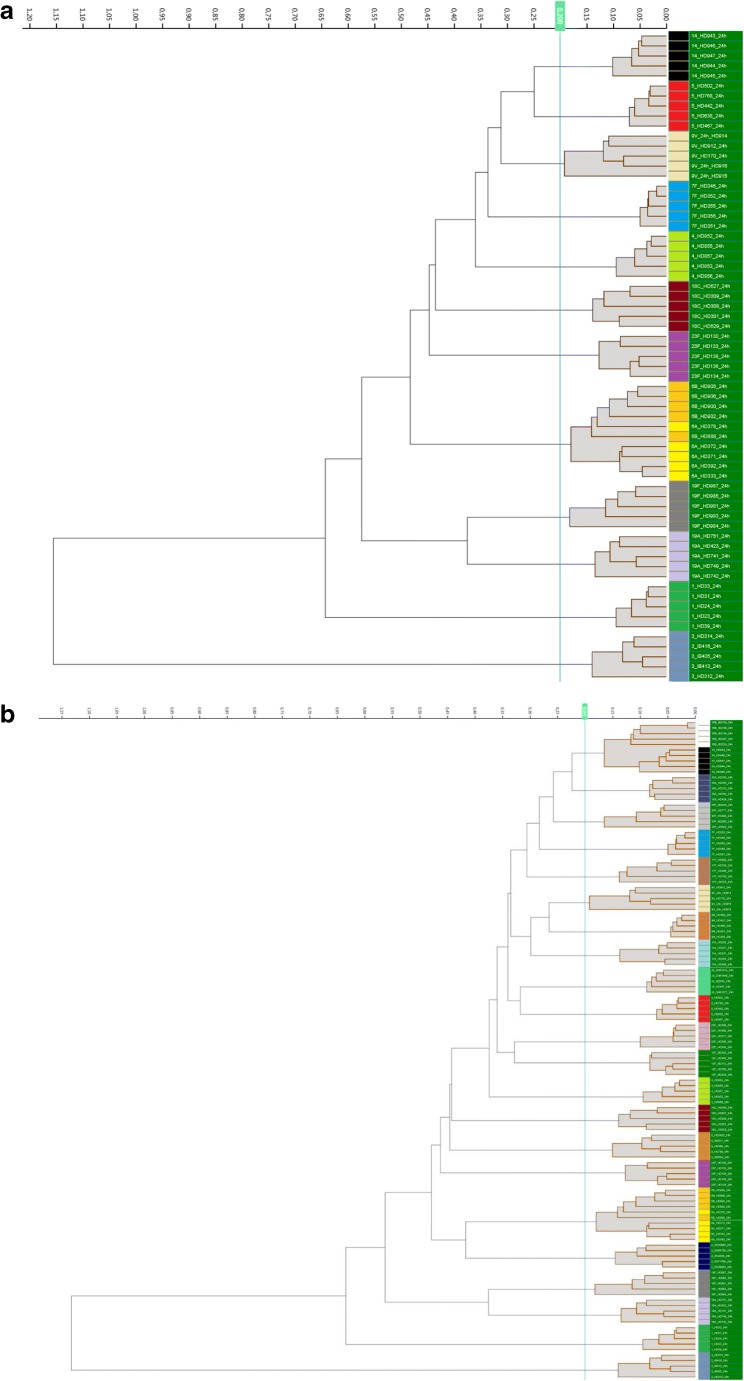
Classification sets for typing/clustering of **a** 13 different serotypes (PCV13 classification set) and **b** 24 different serotypes (PSV23 classification set); strains cluster according to their serotype with the exception of serotype 6A and B; to improve discernibility within the tree, spaces between strains clustering together with a cut-off of less than 0.2 are filled with very light grey; individual strains are colour-coded according to their serotype: serotype 14: black, serotype 5: red, serotype 9V: ochre, serotype 7F: blue, serotype 4: light green, serotype 18C: dark brown, serotype 23F: magenta, serotype 6B: light orange, serotype 6A: yellow, serotype 19F: grey, serotype 19A: light purple, serotype 1: dark green, serotype 3: light blue, serotype 15B: white, serotype 10A: cobalt blue, serotype 33F: light grey, serotype 17F: light brown, serotype 9N: rose, serotype 11A: light blue, serotype 22F: light rose, serotype 20: neon green, serotype 12F: dark green, serotype 2: dark blue, serotype 8: orange

For clustering/typing of a challenge strain, a single mean spectrum of the challenge strain was added to the respective classification set (PCV13 or PSV23). This resulted in either a match or no-match with a pre-existing cluster. 0.2 was used as cut-off for matching. 0.2 was chosen as cut-off, because in both classification sets, this resulted in a good discrimination between clusters (see Fig. 1a and b). A higher cut-off would have resulted in combined clusters; a lower cut-off would have divided clusters.

### Statistical analysis

We compared the observed agreement between the two methods (Quellung reaction and FT-IRS) with the expected agreement by chance alone using the kappa statistic in Stata Software Release 12. Kappa values greater than 0.75 were rated as representing excellent agreement and values from 0.4–0.75 were rated as fair to good agreement [21].

## Results

A total of 120 pneumococcal strains covering 24 different serotypes were used to create a database containing the infrared spectra of all PCV13 and PSV23 serotypes. For data analysis, we used two sets of spectra. Classification set 1 (“PCV13”) contained all 13 serotypes included in the PCV13 vaccine. Classification set 2 (“PSV23”) contained all 23 serotypes included in the PSV23 vaccine and serotype 6B. Using the Euclidean–average linkage algorithm a hierarchical clustering was performed. The strains clustered along their serotype or serogroup. A cut-off of 0.2 grouped all strains into serotype specific clusters with two exceptions (Fig. 1a and b). FT-IR spectra of serotype 14 and 15B were distinct but only resolved into two separate clusters after reducing the cut-off to 0.15. Serotype 6A and B did not form two separate clusters. They formed a mixed joined cluster.

**Fig. 2:**
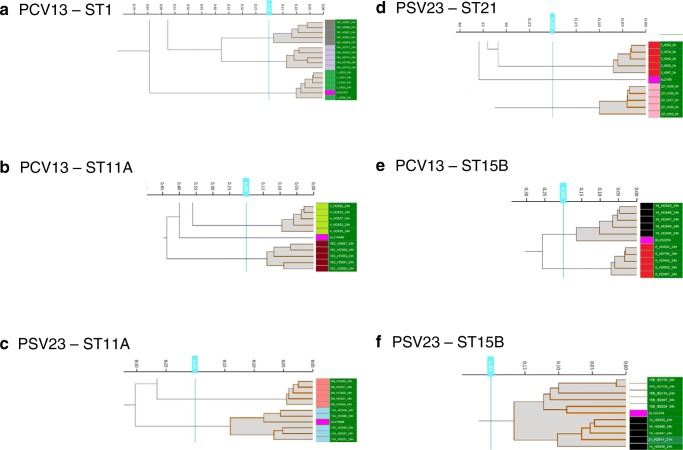
Classification sets for typing/clustering (PCV13, PSV23 classification set) with challenge strains: challenge strains are pink; cut-off for match or no match: 0.2; serotype colour-codes: see legend Fig. 1. Shown are the decisive sections of the respective classification sets: **a** challenge strain (AL612616): serotype 1, matches with the serotype 1 cluster of the PCV13 classification set; **b** challenge strain (AL218499): serotype 11A, no match within the PCV13 classification set; **c** challenge strain (AL218499): serotype 11A, matches with the serotype 11A cluster of the PSV23 classification set; **d** challenge strain (AL211459): serotype 21, no match within the PSV23 classification set; **e** challenge strain (BL202254): serotype 15B, matches with the serotype 14 cluster of the PCV13 classification set; **f** challenge strain (BL202254): serotype 15B, matches with the serotype 15B (sub-)cluster of the PSV23 classification set

**Fig. 3:**
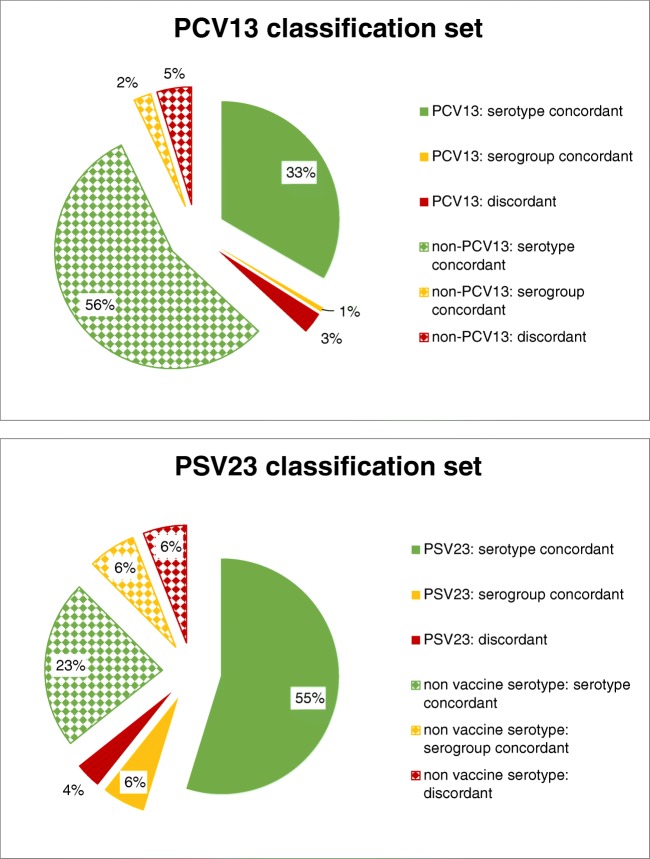
Typing results of 168 challenge strains with PCV13 and PSV23 classification sets; pattern codes: according to serotypes of challenge strains: solid: serotypes of challenge strains are part of the respective classification set; checkerboard: serotypes of challenge strains are not part of the respective classification set; colour codes: according to level of typing agreement between the Quellung reaction and FT-IRS: green: typing result concordant to serotype level between methods; yellow: result concordant to serogroup level; red: discordant results

To predict the serotype of a given strain, both classification sets were challenged with 168 additional strains covering 48 different serotypes: 62 strains with a PCV13 serotype, 46 with a non-PCV13 but PSV23 serotype and 60 with a non-vaccine serotype. A total of 123 isolates were from invasive disease and 45 isolates were from non-invasive disease (Table 1). For an overview of serotypes, see supplementary Table 1. The challenge showed that the spectrum of a given strain mostly fell into the correct serotype cluster as long as spectra of its serotype were part of the classification set (see Fig. 2a–f). Non-classification-set serotypes resulted either in a no-match (see Fig. 2b, d) or a mismatch according to the cut-off (see Fig. 2e). The challenge of the PCV13 classification set with PCV13 serotype strains gave a correct serotype in 56 of 62 (90.3%) cases (see Table 2, Fig. 3 and supplementary Table 1). The serotype 6A strain clustered within the serogroup 6 cluster and 5 strains gave a discordant result (3× serotype 3 (no-match (nm)), 2× serotype 19F (nm)). Of 46 PSV23 serotype strains, 42 (91.3%) clustered correctly. All 4 serotype 15B strains matched with serotype 14. Fifty-two of the 60 non-vaccine-type strains (86.7%) did not match with an existing cluster, that is, they were typed correctly as a serotype not present in the classification set. (These strains are marked as “correct serotype” in Table 2 and supplementary Table 1.) Four strains were assigned a serotype within the correct serogroup (2× 23B (23F), 2× 9A (9V)). Four of six serogroup 15 strains (3x 15A, 1x15C) matched with serotype 14. The challenge of the PSV23 classification set with strains of all 24 different serotypes gave a correct serotype in 92 of 108 (85.2%) cases. Two strains clustered to the correct serogroup (1× 9N (9 V), 1× 6A (serogroup 6)). All 4 serotype 14 strains matched with the combined 14/15B cluster. Using a cut-off of below 0.2 would have clustered them correctly into the serotype 14 sub-cluster. All 4 serotype 15B strains matched with the combined 14/15B cluster. Using a cut-off of below 0.2 would have clustered them correctly into the serotype 15B sub-cluster. If these 8 strains were counted as correct serotype, 100 of 108 (92.6%) would have clustered correctly.

**Table 2:** Classification of challenge strains with the PCV13 classification set and the PSV23 classification set (summary); for data stratified according to serotype, see supplementary Table 1

Serotype of challenge strains	Concordance	PCV13 classification set	PSV23 classification set
PCV13	Serotype concordant	56	52
	Serogroup concordant	1	5
	Discordant	5	5
Sum PCV13		62	62
PSV23 (and not PCV13)	Serotype concordant	42	40
	Serogroup concordant	0	5
	discordant	4	1
Sum PSV23 (and not PCV13)		46	46
Non-vaccine serotype	serotype concordant	52	39
	serogroup concordant	4	11
	discordant	4	10
Sum non-vaccine serotype		60	60
Total		168	168

Six strains showed a discordant result (33F (10A), 2× 19F (nm), 3 × 3 (nm)). One serotype 19F strain would have clustered correctly if a cut-off of 0.22 had been used. Of 60 strains with a non-vaccine serotype, 39 (65%) were correctly assigned a no-match (nm). Eleven strains were assigned to a serotype of the correct serogroup (1× 12B (12F), 3× 15A (15B), 2× 15C (15B), 2× 23B (23F), 1× 33A (33F), 2× 9A (9 V)). Ten strains were incorrectly assigned to be serotype 10A strains (1× 11B, 1× 15A, 1× 23A, 2× 35B, 4× 35F, 1× 38).

The calculation of kappa as a measure for method agreement gave a value of 0.83 for the PCV13 classification set and a value of 0.75 for the PSV23classification set. If matching of serotype 14 and serotype 15B strains was considered to be correct, kappa would increase to 0.77.

## Discussion

The reference method for capsular typing of pneumococci is the Quellung reaction (i.e. serotyping). The initial description of the principle of the Quellung reaction was published by Fred Neufeld in 1902 [22] and still is in use today [23]. To determine a serotype using the Quellung reaction requires more than 100 different pool and type, group and factor sera. These sera are generated by immunization of white rabbits with purified capsular polysaccharides. Subsequently specific factor sera are generated by absorption, i.e. cross-reacting antibodies are eliminated by exposure to cross-reacting antigens (e.g. to distinguish between serotype 6A and 6B). Pool, type and group sera are generated by pooling sera of the necessary specificities. Due to the amount of consumables, this method is only available at reference centres.

FT-IRS as a method for capsular typing was previously described for 6 different bacterial species including *S. pneumoniae* [24]. For *S. pneumoniae*, typing was only done retrospectively, that is for strains with known serotype from a strain collection. Typing was not done prospectively [16].

In our study, 150 of 168 (89.2%) challenge strains were correctly typed to serotype level using the PCV13 classification set and 130 of 168 (77.4%) challenge strains were correctly typed to serotype level using the PSV23 classification set. All kappa values were equal or greater than 0.75 and indicated an excellent agreement between the two methods [21].

The discordant typing results fell within two groups: (A) the correct result should have been assigned because the challenge serotype was in the classification set and (B) the correct result should have been a “no-match” because the challenge serotype was not part of the classification set. With the PCV13 classification set, 5 strains fell in error category A. One of the two serotype 19F strains with a no match would have fallen within the correct cluster if a cut-off of 0.22 would have been used instead of a 0.2 cut-off. We have no explanation why the second strain did not cluster correctly. The three serotype 3 strains, which showed a no-match, were remarkable in several aspects. They were all isolated in 2018, they were serotype 3 strains according to PCR and the Quellung reaction and they were isolated from patients with invasive and non-invasive disease. All serotype 3 strains grew mucoid on blood agar. However, during the application of these three serotype 3 strains to the target, we had the impression that the viscosity of the mucus was different. This might be the reason for the no match when using FT-IRS. The serotype 6A strain was assigned to serogroup 6 because serogroup six cannot be resolved with the current FT-IR spectra acquisition method and clustering scheme. This is not an error per se but points at the limit of the current analysis method. The difference between the capsules of serotype 6A and B is the bonding between the monomers rhamnose and ribitol (6A: 1➔3, 6B: 1➔4) not a difference in monomers. In contrast, serotype 6C can be clearly distinguished from the 6A/B cluster. Compared with the capsule of serotype 6A, the capsule of serotype 6C contains glucose instead of galactose [10]. Preliminary experiments with further refined hierarchical clustering methods showed promising results for the differentiation of serotype 6A and 6B. Further testing with more strains will be necessary to improve the method in this aspect.

Of all non-PCV13 serotypes/strains tested with the PCV13 classification, set 4 strains were assigned a serotype despite the fact that the true challenge serotype was not present in the PCV13 classification set. Two 23B strains were assigned a 23F serotype and two serotype 9A strains were assigned a serotype 9V. The problem to distinguish between members of serogroup 23 was already described [16]. However, we are the first to describe problems within serogroup 9. Looking at the data with the PSV23 classification set revealed that error types A) and B) occurred. Interestingly, as long as at least one member of the same serogroup was present in the dataset, some strains showed a match with the serogroup member instead of giving a no-match (e.g. 23B➔23F). Serotype 38, 11B, 15A, 23A, 35B and 35F strains showed true mismatches. The mismatch assigned was always serotype 10A. To further evaluate this striking result, we added spectra of other serotype 38, 11B, 15A, 23A, 35B, 35F strains to the database and created a third classification set containing these serotypes and serotype 10A. A hierarchical clustering showed that the FT-IR spectra of these serotypes were all very closely related (data not shown). Most importantly, the strains still clustered according to their serotype with a cut-off of 0.16. A challenge of this third classification dataset with spectra of the initial challenge strains showed a correct typing for nine out of ten strains.

FT-IRS and Quellung reaction examine the same structure of the pneumococci, i.e. the capsular polysaccharides, but instead of using epitope-specific binding by antibodies, FT-IRS results depend on the chemical composition of the capsule because IR light absorption is dependent on the chemical structure and their particular molecular bonds. This means the Quellung reaction is directly correlated to the 3-dimensional conformation of the capsular polysaccharides whereas FT-IRS depends on the individual bonds within the pneumococcal capsule. Therefore, it is very likely that some differences in the structure and composition of the capsule are visible in FT-IRS but not with the Quellung reaction (e.g. serotype 3) and vice versa (serogroup 6).

Our study has several limitations. First, we only used isolates from invasive disease for database generation and classification dataset definition. This is because within the last years, the main focus of pneumococcal research was invasive pneumococcal disease. However, prediction of serotype was tried with strains isolated from invasive and non-invasive disease. Second, not all currently described serotypes are present in the database. We restricted our database to the serotypes contained in the currently available vaccines. The aim of this study was to evaluate the FT-IRS technique for fundamental suitability for capsular typing, i.e. this study was organized as a proof of principle study and not meant to present a ready to use substitute for the Quellung reaction. Addition of all serotypes to the database is currently an ongoing project.

A consequence of the restriction of the database to vaccine serotypes is that we now can determine with FT-IRS whether a pneumococcal disease in an actual patient was caused by a vaccine or non-vaccine serotype, i.e. whether the disease was potentially vaccine preventable or not. Patients and physicians can be informed during the actual hospital stay and no follow-up in the outpatient department is necessary. The serotype determination can be done on site within 24–48 h after isolation and identification of a pneumococcus from a patient sample. Most importantly, this short time to result will enable us to follow-up on reports that the serotype itself is an important factor in diseases outcome [7–9]. If prospective and randomized clinical studies can show that the serotype of *S. pneumoniae* is clearly associated with the course of disease, this will impact therapeutic strategies. The short time to result of FT-IRS is the important prerequisite to undertake such clinical studies and finally to include the serotype of *S. pneumoniae* into an individualized therapy.

Finally, we would like to mention that in our opinion, FT-IRS does not only hold a huge promise for capsular typing of pneumococci. We think that it would be worthwhile trying to use FT-IRS for typing of other encapsulated species, for example *Haemophilus influenzae*, meningococci or even *Klebsiella* spp.

## Electronic supplementary material

Supplementary table 1 (XLSX 18 kb)

## Abbreviations

PCV13: 13-valent pneumococcal conjugated vaccine
PSV23: 23-valent pneumococcal polysaccharide vaccine
GNRCS: German National Reference Centre for Streptococci, Aachen, Germany
FT-IRS: Fourier-transformed infra-red spectroscopy
